# Task-irrelevant financial losses inhibit the removal of information from working memory

**DOI:** 10.1038/s41598-018-36826-x

**Published:** 2019-02-08

**Authors:** Sean James Fallon, Nina Dolfen, Francesca Parolo, Nahid Zokaei, Masud Husain

**Affiliations:** 1 Department of Experimental Psychology, University of Oxford, Oxford, UK; 2 Nuffield Department of Clinical Neurosciences, John Radcliffe Hospital, Oxford, UK; 3 Oxford Centre for Human Brain Activity, Wellcome Centre for Integrative Neuroimaging, Department of Psychiatry, University of Oxford, Oxford, UK; 4 Motor Control & Neuroplasticity Research Group, KU Leuven, Leuven, Belgium

## Abstract

The receipt of financial rewards or penalties - though task-irrelevant - may exert an obligatory effect on manipulating items in working memory (WM) by constraining a forthcoming shift in attention or reinforcing attentional shifts that have previously occurred. Here, we adjudicate between these two hypotheses by varying – after encoding- the order in which task-irrelevant financial outcomes and cues indicating which items need to be retained in memory are presented (so called retrocues). We employed a “what-is-where” design that allowed for the fractionation of WM recall into separate components: identification, precision and binding (between location and identity). Principally, valence-dependent effects were observed only for precision and binding, but only when outcomes were presented *before*, rather than after, the retrocue. Specifically, task-irrelevant financial losses presented before the retrocue caused a systematic breakdown in binding (misbinding), whereby the features of cued and non-cued memoranda became confused, i.e., the features that made up relevant memoranda were displaced by those of non-cued (irrelevant) items. A control experiment, in which outcomes but no cues were presented, failed to produce the same effects, indicating that the inclusion of retrocues were necessary for generating this effect. These results show that the receipt of financial penalties – even when uncoupled to performance – can prevent irrelevant information from being effectively pruned from WM. These results illustrate the importance of reward-related processing to controlling the contents of WM.

## Introduction

Efficiently controlling the contents of working memory (WM) is crucial given its capacity limits. One of the ways this can be achieved is to prioritise the storage of currently relevant information^1,2^. Indeed, directly informing participants about the relevance of items currently held in WM, for example by presenting a cue either before (pre-cue) or after (retrocue) encoding can substantially improve recall for cued items at the expense of non-cued items^3–5^. Although these cues can overcome the limitations of WM, in the real world, people are rarely told what items are relevant. Instead, people must discern the relevance of information through their own experience. The mechanisms that enable this process to occur have rarely been included in classic models of WM^6^.

**Figure 1:**
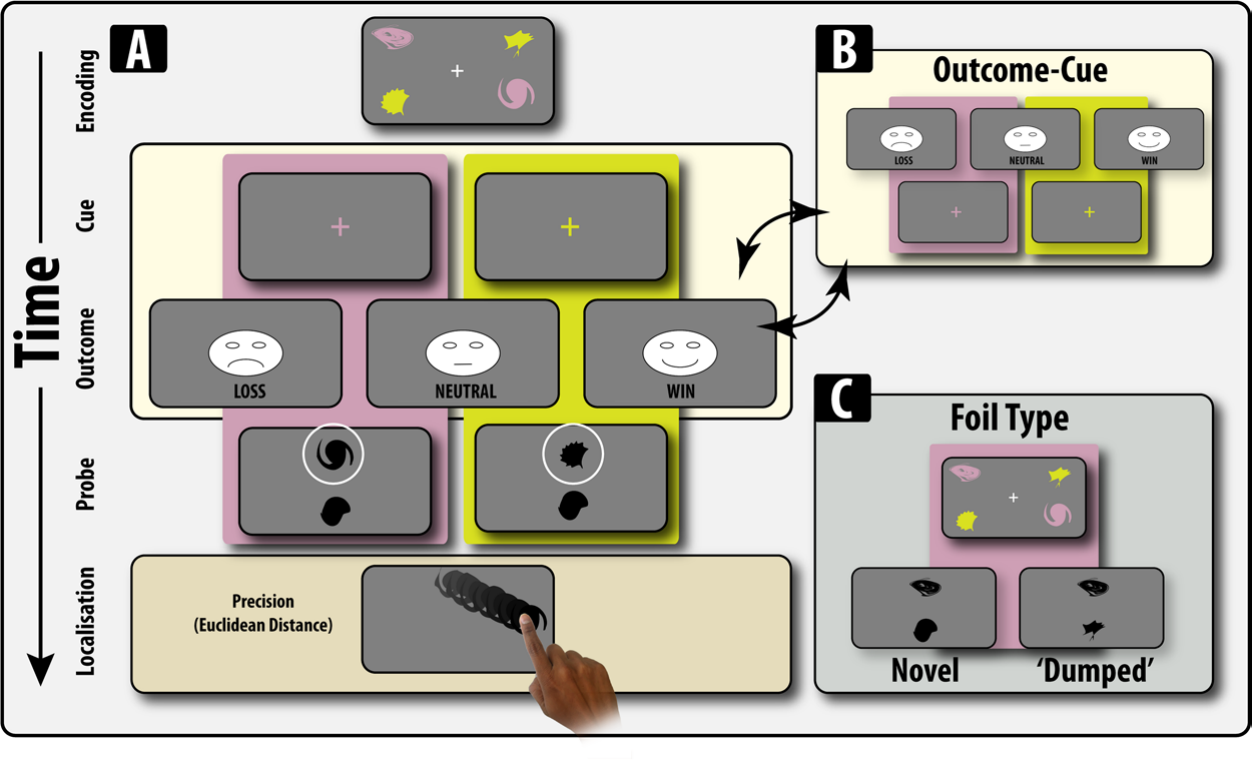
Experimental Task Design Participants were initially presented 4 stimuli in two different colours for 2000ms. After a delay, participants were either presented with a retrocue and one of three unexpected financial outcomes. In the cue-outcome condition (shown), participants were first presented a retrocue, the colour of which indicated what items needed to be retained. For example, if a yellow coloured cue was presented, participants had to remember ONLY the yellow shapes. Following this, participants were presented with 1 of three financial outcomes (unrelated to performance). Presentation of a loss or win indicated that participants either lost or won £1 respectively. A neutral condition indicated that participants neither won nor lost money. In contrast to the cue-outcome condition, in the outcome-cue conditions the order of these two events was reversed. Participants’ memory was probed by having to identify which of the two shapes matched the shape of one of the target shapes (indicated by the colour cue). In half of the trials the foil shapes presented next to the target where novel, in the other half of trials the shapes were drawn from one of the non-cued shapes. For example, in the above example, if the cue was yellow the distracter items would be one of the pink shapes. After identification, participants were required to drag the item to the location in which they remember it being presented.

It has been hypothesised that reward- or punishment-related signals can be used as such a mechanism, and guide the selection and retention of information in WM in a similar way to explicit cues by modulating attention^7–9^. Previous demonstrations have shown that receipt of reward-related information, even though task-irrelevant and unrelated to performance, can modulate WM by changing the neural response in areas responsible for the inhibition of forthcoming distracters and their coupling with reward-related regions such as the ventral striatum^10^. Thus demonstrating that the receipt of task-irrelevant financial information undergoes obligatory processing that can affect WM. The results from this study could be taken to suggest that task-irrelevant financial outcomes (gains and losses) alter WM representations in a proactive fashion, and potentiate the response to future stimuli that need to be gated into, or out of, WM. However, because the reward-related information was interspersed between the presentation of two pairs of stimuli it is difficult to ascertain whether reward-related guidance of WM acts proactively to potentiate attentional manipulations of mnemonic stimuli or consolidates manipulations (attentional shifts) that have already occurred. In contrast, ideas stemming from the learning literature suggest an alternative hypothesis: the receipt of gains or losses exert their effects retroactively and serve to promote or prohibit changes in attention that are correlated with these events^11^. For example, decades of research on attention has shown that reward and punishment can be used to generate and sculpt attentional sets, strengthening the representations of attended items^12–16^. These studies suggest that reward-related information can consolidate or reinforce attentional shifts that have already occurred. Here, we sought to adjudicate between these two hypotheses. Critically, we sought to eliminate the effect that reward-related information may have on the initial encoding of memoranda by using a retrocue design in order to manipulate the contents of WM. In this study, after the encoding of stimuli, we varied whether value signals (of different valence) were delivered before (potentiate) or after (consolidate) the presentation of a cue (retrocue) that directed attention to be shifted to a subset of the encoded items stored in WM (Fig. 1).

The way in which WM recall is measured can occlude our view of its architecture^8,17^. A crucial feature of our approach is the way in which we probe recall. Previous studies that have assessed the role of financial outcomes on WM have used traditional binary report measures – in which participants are probed by being asked to make a binary judgement upon whether a probe image was or was not part of the encoded items^10^. More recently derived methods have also sought to examine the quality of recall by asking participants to reproduce the exact features of memoranda^18–21^. Here, we exploit a previously used probe method “What-is-Where”^19^, in which, after having encoded a set of items in different spatial locations, participants are not only asked to identify memoranda, but also to reproduce a feature of that item, i.e., its spatial location. In addition to enhanced sensitivity, we can use this method to understand *how* reward-related processing can cause WM recall to go awry. For example, although an item may be correctly identified, other features or properties of that item, such as its spatial location, can become corrupted with features from other items. These events can be labelled as misbinding errors^19,22^.

In this experiment, we examine the effect of varying the presentation of value signals before or after a retrocue. Participants were presented with an array of four-items in two separate colours that had to be encoded. In one condition, participants were presented with a retrocue which indicated that only a subset of the memoranda needed to be retained for future recall. Following this, they were presented with a task-irrelevant financial outcome (loss, neutral or gain). Alternatively, in the other condition, the order of the retrocue and task-irrelevant outcome was reversed (Fig. 1). We assessed their memory for identity, location and their binding. However, in order to parse the effects of retrocuing from outcome receipt, we also included a control condition in which outcomes were presented as in the experimental versions, but there was no retrocue presented.

## Results

### Re-presentation of dumped items impairs accuracy

Firstly, we examined identification accuracy, which is the ability to correctly identify the target shape from either a novel foil or a distracter foil (one of the non-cued shapes). The valence of the task-irrelevant outcomes did not significantly alter identification accuracy (*F* < 1), neither did it significantly interact with any other variable (all p’s >0.141). The order of the cue and outcome significantly affected accuracy (*F*(1,51) = 6.71, *p* = 0.012), with higher accuracy when the cue preceded the outcome phase compared to outcome preceding the cue phase. Re-presenting the dumped items as foils, compared to novel items, led to significantly impaired identification accuracy (*F*(1,51) = 8.35, *p* = 0.006) and there was a trend for this effect to vary according to outcome-cue order, (*F*(1,51) = 3.53, *p* = 0.066). Thus, in summary, the ability to correctly identify items was not affected by the valence of the task-irrelevant financial outcome, irrespective of the cue-outcome order.

### Losses impair the precision of WM representations but only when presented before retrocues

Next, we examined the precision of mental representations by looking at the discrepancy between the location that the correctly chosen memoranda was dragged to and its presented location. There was a significant main effect of order, with greater error on the outcome-cue compared to cue-outcome (*F*(1,51) = 4.26, *p* = 0.044). However, there was a significant interaction between valence and order (F(2,102) = 4.17, p = 0.018), due to there being no significant effect of valence in the Cue-Outcome condition (*F* < 1), but there was a significant effect in the outcome-cue condition (*F*(2,48) = 3.53, *p* = 0.037). This significant effect was due to error being higher on loss trials compared to both neutral (*t*(26) = 2.65, *p = *0.014) and win trials (*t*(26) = 2.19, *p* = 0.038), but there was no significant difference between neutral and win trials (*t*(26) = 0.17, *p* = 0.86). There was no significant interaction between valence and probe type (*F*(2,102) = 2.05, *p* = 0.134) and none of the other effects were significant (*F* < 1). Thus, with regards to precision, these results suggest that valence-specific effects of task-irrelevant information only occur when presented *before*, but not after the retrocue.

### Losses presented before the retrocue increase misbinding to the dumped items

Next, we sought to further specify the nature of this localisation problem according to outcome valence by examining misbinding to non-targets (dragging the target item to non-target locations, see methods). There was no main effect of valence (*F* < 1), but valence did significantly interact with cue order (*F*(1,102) = 3.81, *p* = 0.025). The same pattern as was observed for the localisation performance was also observed for misbinding, losses compared to wins significantly increased misbinding in the outcome-cue condition (*p* = 0.036). No other effects were significant (*p*’s > 0.14).

A further question that can be asked is *which* location participants erroneously report as the target location when they make their localisation responses: the location of the non-probed cued (target) item or the non-cued (irrelevant item).

No such interaction between cue order and valence was observed for misbinding to the other non-probed target (F < 1). However, for misbinding to non-target (irrelevant) items, there was an interaction between cue order and valence in influencing misbinding (*F*(2,102) = 5.29, *p* = 0.006). Again, this was due to losses increasing misbinding compared to wins (*p* = 0.002) and, here, losses also increased misbinding compared to neutral trials (*p* = 0.034). No other effects were significant (*p*’s > 0.164). Thus, in summary, receiving task-irrelevant financial outcomes affected binding between items and their locations. Losses increased misbinding only in the condition where this outcome was received prior to the retrocue (outcome-cue). This misbinding was also only prominent when examining misbinding to the non-cued (irrelevant items).

### Control Experiment

We performed a further control experiment in order to examine the role of the retrocue and mnemonic load in producing the above results. Accordingly, we conducted an experiment that was identical to the Outcome-cue condition, except that no retrocue was presented, i.e., only outcomes of different valences were presented after encoding. Also, to examine load effects, we varied whether two or four items were presented at encoding. The timings between encoding and probe were the same as those for the Outcome-cue condition. Accuracy, precision and misbinding were analysed using repeated measures ANOVA with set size and valence as within-subject factors.

For accuracy, participants were significantly more accurate for low (two shape) compared to high (four shapes) load trials (*F*(1,26) = 155.02, *p* < 0.001), but there were no effects of valence or interaction between setsize and valence (*F*s < 1).

Precision and misbinding were both increased for high compared to low set sizes (precision, *F*(1,26) = 345.449, *p* < 0.001; misbinding, *F*(1,26) = 769.01, *p* < 0.001). For neither measure was there any significant main effects of valence (*F*s < 1). A significant interaction between set size and valence was observed for precision (*F(*2,52) = 3.40, *p* = 0.041) and a near significant interaction for misbinding (*F*(2,52) = 3.00, *p* = 0.058). Crucially, however, these interactions were due to valence affecting precision and misbinding at low loads (*F*(2,52) = 5.35, *p* = 0.008 for precision; *F*(2,52) = 3.22, *p* = 0.48 for misbinding) and not high loads (*Fs* < 1). This was due to win trials causing both higher localisation error (*t*(25) = 3.17, *p* = 0.004) and misbinding errors (*t*(25) = 2.51, *p* = 0.018). None of the other pairwise comparisons were significant (all p’s > 0.073). Thus, these results suggest that merely having four items to remember at the point of outcome receipt, as when the outcome precedes the retrocue, does not produce the effects on precision and misbinding seen in the Outcome-Cue condition (Fig. 5). However, impairing effects of outcome were observed in the two-item condition for precision.

## Discussion

The results of this study suggest that financial outcomes – though completely irrelevant to the task and uncoupled to performance – can influence the ability to respond to a cue indicating that information can be removed from WM. A control experiment revealed that this difference was not due to differences in load at the point of the receipt of financial outcomes. Rather, qualitatively different results were observed when no retrocue was presented, but only on low load trials. Contrary to intuitive ideas suggesting that financial rewards promote generalised cognitive improvements, across the retrocue studies, value signals only affected WM when presented prior to a retrocue (Figs 3 and 4). Specifically, the presentation of task-irrelevant financial losses increased misbinding. This effect was particularly prominent when examining the level of misbinding (mislocalisation) between the probed item and the irrelevant items that should have been jettisoned from memory **(**Fig. 4B). The finding that participants are more likely to misbind to items that should have been purged from WM suggest that loss-related signals modulate the extent to which irrelevant information can be removed from WM or prevented from exerting an interfering effect. In contrast, in the absence of a retrocue and at low loads, receipt of financial rewards actually *decreased* precision and *increased* misbinding (Fig. 5B,C). This effect is potentially a qualitatively distinct process to that observed in the retrocue experiment and may result from confusing the features, i.e., spatial location, of relevant information.

### How does reward-related processing impact on WM?

**Figure 2:**
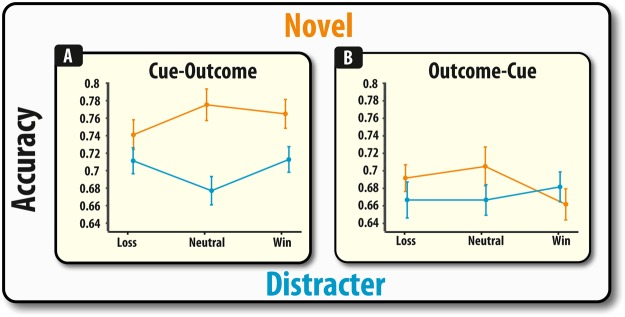
Accuracy depicted as a function of outcome for the Cue-Outcome (panel A), Outcome-Cue (panel B). Error bars represent within (demeaned) errors bars. Asterisks indicate significant pairwise group differences (p < 0.05; see main text for details).

**Figure 3:**
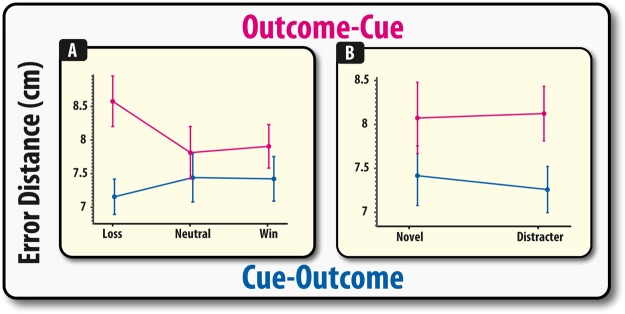
Misbinding towards the non-probed target (panel A) and non-cued items (panel B) depicted as a function of outcome for the Cue-Outcome and Outcome-Cue conditions. Error bars represent SEM. Asterisks indicate significant pairwise group differences (p < 0.05; see main text for details).

**Figure 4:**
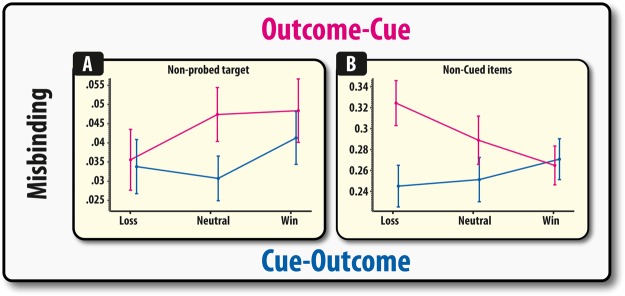
Misbinding towards the non-probed target (panel A) and non-cued items (panel B) depicted as a function of outcome for the Cue-Outcome and Outcome-Cue conditions. Error bars represent SEM. Asterisks indicate significant pairwise group differences (p < 0.05; see main text for details).

A variety of studies have found that reward can improve WM recall and its neural representations^23–27^. Congruent with these findings, it has been proposed that reward-related signals can affect WM representations in a valence-specific manner with receipt of positive outcomes strengthening the neurocognitive mechanisms of distracter-resistance, whereas negative outcomes increase the effect of distracting information^10^. Thus, the present results are in accordance with previous reports that financial outcomes – although task-irrelevant - can exert effects that can modulate memory in a context-dependent manner. As suggested by previous studies, this obligatory effect of task-irrelevant financial outcomes may stem from the fact that reward-related processing and WM share, or act upon, common neuroanatomical substrates^1,7^. These studies, however, were unable to comment upon the exact psychological mechanisms that were responsible for generating these effects, i.e., it was unclear from these studies how reward-related signalling was affecting WM representations as they used a simple binary measure of WM recall. Here, by using an analogue measure of probing WM, the precise effect of task-irrelevant financial outcomes can be specified. The receipt of task-irrelevant financial losses was not found to affect the identification of relevant information (Fig. 2), but rather the precision with which items were remembered (Fig. 3) – an effect likely to be attributable to increased misbinding (Fig. 4).

**Figure 5:**
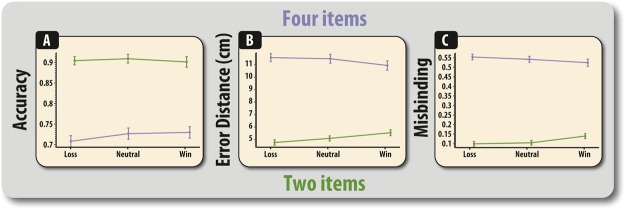
Performance on the control task for two and four item trials for identification accuracy (**A**), localisation performance (**B**) and misbinding (**C**). Error bars reflect within-subject standard error of the mean.

The effect of task-irrelevant financial outcomes on WM binding was found to be heavily dependent upon whether the value signals were presented before or after the need to shift attention in WM (Fig. 3) and whether any retrocues were indeed presented (Fig. 5). The neural substrates used to represent information has been found to be dependent upon whether attention is directed at that information, i.e., its attentional state^28^. Behaviorally, shifting attention within items stored in WM has been found to confer advantages on the focused items at the expense of the non-focused items^2,5,28^. Thus, information may be in a different neural and psychological state after it has been focused on, which in this experiment was induced by the presentation of a retrocue. The influence of non-cued information on WM has been harder to fully specify. It has previously been shown that items outside the focus of attentional can be “reactivated”, suggesting that this information is not completely removed from memory^29–32^. This implies that information, though not cued, can continue to exist in some form and influence subsequent responding.

The data appear to suggest that retro-cueing participants did not lead to a complete removal of the irrelevant, non-cued, information in this paradigm, and that there are differential effects of these manipulations on identification and precision. Participants made significantly more identification errors on trials where the target had to be selected from amongst one of the non-cued (distracter) items (Fig. 2A,B). Thus, this suggests that irrelevant information, even though participants are 100% certain that they will not be probed on these items, continues to linger in the mind and exert disruptive effects on WM identification. In contrast, there were no significant effects of foil type on precision (Fig. 3B). Instead, the deleterious effect of spatial information appeared to be modulated by task-irrelevant financial outcomes, in both cued (Fig. 3) and non-cued **(**Fig. 5**)** scenarios. Thus, this finding supports previous studies that have found object-based and spatial WM to be subject to different types of degeneration (see^22,33–36^) and reinforces long-standing arguments that these are two separate systems^37,38^.

### Mechanisms through which loss can impair binding

The lingering effects of irrelevant information may also be crucial for understanding the nature of the valence-dependent effects on misbinding. Valence-dependent effects on the levels of misbinding prior to a retrocue were only found to exist when examining the proportion of trials in which participants erroneously dragged the target item to the location of one of the irrelevant (non-cued items), i.e., those items that should have been completely removed from WM due to the retrocue. There were no such effects on misbinding to non-probed targets (those items that participants were still maintaining as possible targets but were not in fact probed). Thus, the key psychological mechanism that seems to be responsible for the loss-induced impairment of WM prior to a retrocue is the confusion between the features of relevant (cued) and irrelevant information (non-cued). This conclusion is further strengthened by the fact that, in the absence of a retrocue, losses did not increase misbinding when remembering four items (Fig. 5C), i.e., when the mnemonic load was similar to that in the Outcome-Cue condition. Thus, it is not the case that the differential effect of outcome valence on misbinding in the retrocue experiments were due to discrepancies in load.

Why unexpected losses should exert this effect is unclear. It could be hypothesised that the enhanced misbinding after unexpected losses in the outcome-followed-by-retrocue condition occurs because losses prevent the non-cued information from being effectively jettisoned from working memory. Unexpected financial losses may exert this effect due to impairing the ability of top-down control to remove irrelevant information. This may be because task-irrelevant financial outcomes exert an obligatory effect on the activation of distracter-resistance networks^10^, with gains improving and losses diminishing this network. Under this framework, financial losses prevent, whereas gains potentiate, the ability to prune irrelevant information after a retrocue and cause distortions in recall.

### Mechanisms through which gains impair binding in the absence of a retrocue

In the absence of a retrocue, task-irrelevant financial gains were found to increase misbinding, but only in low (two-item) and not high (four item) load conditions. Given that only two items were presented, the misbinding events that occurred could only have been to the relevant, but-not-cued-item. This suggests that in the absence of a retrocue, task-irrelevant financial rewards impair WM through allowing the features (spatial position) of relevant information to become confused with each other. The appearance of this effect in this context could not have necessarily been anticipated based on previous work and further studies will be needed to uncover the mechanisms of this effect. However, more broadly, this result chimes with other work highlighting the “downside” of financial gains in impairing specific cognitive functions^39^. Thus, the introduction of financial incentives should not be seen as a means of uniformly improving cognitive control.

### Alternative explanations

Despite this, there are several alternative interpretations of the results that need to be discussed. Firstly, because of the valence-specific nature of the results (losses exerted different effects from wins with neutral trials being intermediate), it is very unlikely that the effects of unexpected losses on binding in WM was due this event inducing an alerting response or due to the occurrence of a salient or distracting event.

Secondly, the valence-specific nature of the results also makes it unlikely that the differential effects observed in the cue-reward and reward-cue conditions are due to differences in load.

This explanation is unlikely because, even with the reward-cue condition, there was an effect of valence. Thus, even when keeping load constant, differential effects of valence on binding were observed. Added to this, is the fact that misbinding was found to occur to one of the non-cued items. A pure-load account would predict that misbinding would be spread between all three objects equally. This was not observed (Figure 4AB). Moreover, a further control experiment, revealed that experimentally manipulating the load of items at the point of outcome receipt did not reproduce the effects seen when manipulating load via retrocues. Thus, the presence of retrocues appear to be essential for the genesis of the loss-induced increase in misbinding. Additionally, it could also be argued that one of the reasons for an outcome effect in the Outcome-Cue condition and not the Cue-Outcome condition is because of the temporal disparity in the period between encoding and outcome receipt in both conditions (3500 ms in the Cue-Outcome and 1500 ms in the Outcome-Cue). While the relationship between time (allowing for information to decay or be actively maintained) needs to be actively studied, we do not feel this timing factor is responsible for generating our results. This is because our control condition, which featured the same timing parameters as in the Outcome-Cue condition, we did not observe a similar outcome effect when the mnemonic load at outcome receipt, i.e., four items, was matched.

### Summary

The presentation of retrocues can help to overcome the limitations in WM by allowing finite mnemonic resources to be concentrated on relevant, and away from irrelevant, items. The results of this study suggest that reward-related mechanisms may play a role in controlling the contents of WM, particularly with task-irrelevant financial losses leading to an impaired ability to prune irrelevant information.

## Method

### Participants

A group of 82 healthy subjects between the age of 19 and 31 years (M = 22.6, SD = 3.01) was recruited to take part in the study. Of these subjects, data from two subjects were excluded due to computer failure (one from the cue-outcome [CO] and one from the control condition). 80 subjects were included in the study (28 Cue-Outcome condition, 25 Outcome-Cue (OC) condition and 27 load control condition; see below for task descriptions). All participants had normal or corrected to normal vision. Participants gave written, informed consent and were paid to participate (£10 + max £6 bonus). The study was approved by the Oxford University Medical Sciences Inter-Divisional Research Ethic Committee and was conducted within accordance to local guidelines.

### Design and procedure

All tasks were programmed in Matlab Psychophysics Toolbox version 3^40^ *Retrocue tasks*: A schematic representation of the tasks are shown in Fig. 1. At the start of each trial, two pairs of shapes are displayed on the screen for a duration of 2000 ms (i.e. *encoding phase*). Items within the same pair have the same colour. Participants are instructed to remember the identity and location of the items in the memory array. *The encoding phase* is followed by a 1500 ms blank delay. Next, subjects are presented with one of two possible events for 500 ms: a financial outcome or a cue (i.e. retro-cue). In the OC condition, the outcome precedes the cue with a blank interval of 500 ms in between. In the CO condition, the cue precedes the outcome with a blank interval of 1500 ms in between. The cue indicates to participants which items are the targets, i.e. which items are going to be probed for recall. For example, a cue in colour 1 indicates that the pair of shapes that appeared in colour 1 are the targets. The cue is valid 100% of the time, i.e., the colour indicated was always instructive of which pair has to be remembered. Subjects are also presented with one of three possible financial outcomes. The choice for the design of the cues was based upon previous studies^10^. A “loss” screen features a sad face flanked by “−££” displayed at the four corners of the screen and the presentation of a negative auditory tone. In case of a “win” screen, a happy face is present in the centre flanked by “+££” at the four corners of the screen and a positive auditory tone (cash register). A “neutral” screen can be recognized by a neutral face surrounded by “===” at the four corners and accompanied by a neutral sound (click). Participants are told that they respectively lost £0.50, won £0.50 or stayed on the same amount of money. The neutral outcome is used as a baseline. Importantly, the financial outcome is independent of performance.

After 1500 ms and 500 ms for the OC and CO condition respectively (1500 ms after the presentation of the cue in both conditions), two probes appear on the screen: one above and one below the central fixation. One of these items was drawn from the cued pair (i.e. the targets) and the other one is a foil (non-target). The foil can either be a novel shape subjects have not seen before or a distractor shape (drawn from the non-selected pair of shapes). The distractor is drawn from the pair of shapes that appeared as part of the uncued pair (i.e., if the cue indicated that pair 2 has to be recalled, the distractor is part of pair 1). Distractors and novel foils were equally frequent. Probes stay on the screen until a response is made, with the position of the target and the foil (i.e. top or bottom) counterbalanced across trials. Participants are instructed to touch the target shape and drag it to the location it appeared in during the encoding phase. Participants are free to localize objects from WM in a continuous space using a touch screen^19^. The spacebar has to be pressed to conform their choice. Each response is followed by a feedback screen which displays the targets on their initial location accompanied by the probe on the chosen location. Note, the timing differences between the various phases of the OC and CO conditions were instituted in order to ensure that there was the time between seeing the items during the encoding phase and being probed on those items.

The task lasted approximately 45 minutes and consisted of 4 blocks. Participants completed in total 144 trials, 24 trials for each the six combinations of outcome (1–3) and foil type (1–2). The design is a 2 (order: cue-outcome/outcome-cue) by 3 (outcome: gain/loss/neutral) by 2 foiltype (novel/distractor) factorial design, with order as a between-subjects factor.

*Control task*: In order to separate out the effects of presenting valenced outcomes from retrocuing on WM recall, we also included a control task, in which outcomes (but not retrocues) were presented. Moreover, it could be argued that differential effects of outcomes may be observed in the Cue-Outcome and Outcome-Cue condition, not because of the interactions with retrocues and outcomes, but because the mnemonic load is lower in the former compared to the latter at the point of outcome receipt. That is, at the point of outcome receipt in the Cue-Outcome condition participants should only be maintaining two items at the point of receiving the valence outcomes, whereas in the Outcome-Cue condition, they are maintaining four items at the outcome phase. Therefore, we also manipulated the set size (two or four items) participants had to encode and recall. The timings and procedure for this paradigm were identical to the Outcome-Cue condition (Fig. 1), except that no retrocue was presented. Also, because all the items were relevant at the point of recall as no retrocue was presented, no distracter (non-cued) items were presented at the identification phase. Thus, there were only novel foils in this experiment. The task lasted approximately 45 minutes and consisted of 4 blocks. Participants completed in total 144 trials (48 trials for each outcome valence).

### Stimuli and screen

The mnemonic material consisted of computer generated “Spirographs” composed of different RGB elements (Fallon & Cools, 2014). Stimuli were presented on a grey background. Items were randomly located on the screen and were at a minimal distance (~9.4 cm) from one another. The display size was 51 cm × 29 cm and all items had a visual angle of approximately 2.7 degrees, based on a distance of 42 cm between the participant and the screen.

### Analysis

Data analysis was performed using Statistical Package for the Social Sciences 22.0 (SPSS IBM Corp). All behavioural variables (below) were entered into mixed three-way ANOVAs (cue-outcome order, valence and probetype). Significant interactions were followed up using paired t-tests (Fisher’s least significant difference).

Accuracy for object identification was calculated as the proportion of total trials in which participants identified the target shape. Accuracy data were arcsine transformed [arcsine (√ x)] to conform to parametric assumptions (Howell, 1998).

The precision of working memory was assessed using error distance for target localization. Error distance was quantified as the Euclidian distance between the position on which the target appeared in the encoding phase and the position it is dragged to in the response phase. Only trials where the correct item was selected were included in the analysis.

Systematic errors in WM recall can happen when the features of one stimuli are erroneously combined with another. Here, misbinding can occur where the spatial locations of a target item become confused with other items. To calculate misbinding we examined the proportion of times in which participants dragged the probed target item (on correct trials only) to the location of one of the other items presented. Items were considered to be dragged to the location of another item if they were within a specified distance of that item. Given that this distance should reflect the precision of memory, participants’ mean precision level (error distance) was used as the radius around an item had to be dragged towards in order to count as a misbind. Moreover, the added benefit of this approach is that we can discern which item (out of the three non-probed target items) participants were misbinding to. Critically, here, we can determine whether participants misbind to the non-probed target (the other cued item that participants were supposed to be maintaining but were not tested on) and the ‘dumped’ or irrelevant items (the non-cued items).
